# Periodontal inflammation: Integrating genes and dysbiosis

**DOI:** 10.1111/prd.12267

**Published:** 2019-12-18

**Authors:** Shaoping Zhang, Ning Yu, Roger M. Arce

**Affiliations:** ^1^ Periodontics Department College of Dentistry University of Iowa Iowa City Iowa USA; ^2^ Applied Oral Science Department The Forsyth Institute Cambridge Massachusetts USA; ^3^ Department of Periodontics Dental College of Georgia Augusta University Augusta Georgia USA

**Keywords:** genome‐wide association study (GWAS), inflammation, microbiome, periodontitis, transcriptome

## Abstract

Biofilm bacteria co‐evolve and reach a symbiosis with the host on the gingival surface. The disruption of the homeostatic relationship between plaque bacteria and the host can initiate and promote periodontal disease progression. Recent advances in sequencing technologies allow researchers to profile disease‐associated microbial communities and quantify microbial metabolic activities and host transcriptional responses. In addition to confirming the findings from previous studies, new putative pathogens and novel genes that have not previously been associated with periodontitis, emerge. For example, multiple studies have reported that *Synergistetes* bacteria are associated with periodontitis. Genes involved in epithelial barrier defense were downregulated in periodontitis, while excessive expression of interleukin‐17 was associated with a hyperinflammatory response in periodontitis and with a unique microbial community. Bioinformatics‐enabled gene ontology pathway analyses provide a panoramic view of the bacterial and host activities as they shift from periodontal health to disease. Additionally, host innate factors, such as genetic variants identified by either a candidate‐gene approach or genome‐wide association analyses, have an impact on subgingival bacterial colonization. Transgenic mice carrying candidate genetic variants, or with the deletion of candidate genes mimicking the deleterious loss‐of‐function variant effect, provide experimental evidence validating the biologic relevance of the novel markers associated with the microbial phenotype identified through a statistical approach. Further refinement in bioinformatics, data management approaches, or statistical tools, are required to gain insight into host‐microbe interactions by harmonizing the multidimensional “big” data at the genomic, transcriptional, and proteomic levels.

## 1 INTRODUCTION

The current model of pathogenesis of periodontal disease underlines the complex interactions among plaque bacteria, the host's genetic factors, and acquired environmental stressors.^1^ Bacteria can elicit inflammatory responses in different host cell populations through interactions of pathogen‐associated molecular patterns and pattern recognition receptors. For example, lipopolysaccharide, a prototype of pathogen‐associated molecular patterns from *Escherichia coli*, is a potent inflammation inducer by activating toll‐like receptor‐4, a classic pattern recognition receptor, on the cell surfaces of macrophages or dendritic cells.^2^, ^3^ Other pathogen‐associated molecular patterns, such as the bacterial cell wall component peptidoglycan or flagellin of motile bacteria, can be recognized by toll‐like receptor‐2 and toll‐like receptor‐5 on the host cell surfaces, respectively.^4^, ^5^ In addition, inflammation can also be readily stimulated by activating intracellular pattern recognition receptors such as nucleotide‐binding oligomerization domain‐like receptors and retinoid acid‐inducible gene I‐like receptors.^6^, ^7^ Although bacteria present in the plaque biofilm initiate inflammation, intrinsic host factors and environmental stressors modulate the magnitude, duration, and extent of an inflammatory response. For example, occasionally patients with poor oral hygiene and abundant calculus present inflammation that is mostly confined to the gingiva while the deeper tooth‐supporting structures are rarely affected. This clinical observation is echoed by historical longitudinal studies monitoring the rate of periodontal destruction, which undoubtedly suggests a difference in disease progression in populations with a similar oral hygiene condition.^8^, ^9^ These distinct susceptibilities to periodontitis clearly indicate a genetic component. In addition, smoking, nutrition, medications, and systemic diseases such as diabetes, also modify periodontal disease activity. Therefore, the genetic makeup and the epigenetic program modulated by environmental stimuli determine the periodontal inflammatory response upon challenge by plaque pathogens at the oral mucosal or gingival surface.

A comprehensive mapping of molecular events, such as transcriptome analysis in tissue samples, offers an integrated global overview of biologic process changes in diseases. Several transcriptome studies have elucidated common pathways or molecular regulatory networks that are specific to different states of periodontal diseases.^10^, ^11^, ^12^, ^13^ Other than those signature molecular events, several novel pathways, such as those associated with neural regulation or epithelial barrier defense function, have emerged.^13^, ^14^ These altered biologic activities, which have not been previously characterized in periodontal disease, provide a fresh perspective on understanding periodontal inflammation as a response to plaque bacteria.

Subgingival regions at the root surface or pocket epithelium harbor a favorable environment for microbial colonization. Plaque bacteria employ various strategies to foster a metastable microbial community that is in harmony with local gingival tissues. However, disturbances that disrupt this symbiosis can lead to periodontal disease. A majority of investigators agree that periodontitis is associated with a shift of subgingival microbial composition in plaque biofilm. Prior to the current widely applied 16S ribosomal RNA gene sequencing by microbiologists, culture‐dependent and DNA‐DNA hybridization techniques had already revealed certain compositional and dynamic changes of the subgingival microbial community from health to disease.^15^, ^16^, ^17^ Microbiome analyses enabled by 16S ribosomal RNA sequencing have not only confirmed the major findings from those classical studies, but have expanded our understanding of the bacterial etiology of periodontal disease by discovering novel taxonomic bacteria that were not previously associated with periodontitis.^18^, ^19^ Information from this ribosomal homology‐based high‐throughput sequencing technique and whole genomic sequencing with an even more refined resolution at the species level provide a global snapshot of bacterial diversities and community dynamics associated with periodontal health and disease conditions.

Similarly to other common polygenic diseases, such as diabetes, coronary disease, or hypertension, the susceptibility to periodontal disease can be explained by the “common disease common variant” hypothesis. In this hypothetic model, disease‐associating genetic variants or single nucleotide polymorphisms present in the coding, regulatory, or intergenic sequences of genes are relatively common in the general population.^20^, ^21^, ^22^ The disease phenotype is determined by the totality of variants, each of which contributes to the clinical disease in varying degrees. However, the effect size of those disease‐associating single nucleotide polymorphisms is usually moderate compared with rare alleles found in rare familial disorders with high penetrance and more severe clinical disease presentations.^22^ Genome‐wide association analysis represents an unbiased discovery tool to identify disease trait‐associating genetic variants. Several genome‐wide association studies have uncovered a number of candidate genetic loci or haplotypes that are significantly associated with different clinical and biologic periodontal inflammatory phenotypes at the genomic scale.^23^, ^24^, ^25^, ^26^, ^27^ Many of these disease‐associated genomic signals exist in or approximate to genes that have an unknown function or have not been previously implicated in periodontitis.

Genetic variants also have an impact on the colonization of bacteria in biofilm. Two models have been proposed to explain this genetics‐associated dysbiosis.^28^ In the first model, single nucleotide polymorphism variants may compromise genes that are associated with pathways of bacterial sensing and recognition. For example, variants in the promoter or coding sequence of pattern recognition receptor genes, or genes encoding binding partners of pattern recognition receptors in the downstream signaling events, would clearly affect the colonizing capacities of certain bacteria in plaque biofilm. In the second model, the variant‐affected genes lead to an excessive inflammatory environment in the subgingival niche, which favors the growth of specific biofilm bacteria. The increased production of gingival crevicular fluid caused by a hyperinflammatory response provides nutrients for plaque bacteria. Indeed, the inflammation‐associated metabolic changes of bacteria can influence the mass and structure of subgingival biofilm. In addition, tissue breakdown caused by an excessive inflammatory response also feeds pathogenic bacteria and affects biofilm formation.^29^

In this paper, we review the recent advances gained from periodontal “‐omic” studies that profile host‐bacteria interactions with a global panoramic overview. We summarize the molecular mechanisms of the inflammatory response in periodontal transcriptiome studies, the changes in diversity and dynamics of subgingival microbial flora, and the host genetic underpinning of bacterial colonization in plaque.

## 2 FINDINGS OF TRANSCRIPTOME STUDIES IN GINGIVITIS AND PERIODONTITIS

With the improved resolution of sequencing technology that has become more readily available, transcriptome data provide a genome‐wide atlas of altered gene expression associated with periodontal disease processes. Several studies aiming to explore the distinct gene expression profiles and pathways unique to periodontitis have indeed confirmed the anticipatory upregulation or downregulation of genes involved in inflammation or bone resorption.^14^, ^30^ Examining aggressive and chronic periodontitis gingival biopsy tissues through gene ontology, Demmer et al^14^ identified that p38, c‐Jun N‐terminal kinase, and mitogen‐activated protein kinase pathways were significantly upregulated in diseased tissues, while several members of the transforming growth factor‐beta family were downregulated in diseased tissues compared with disease‐free control samples. In addition, they also reported upregulation for a majority of chemokines, platelet‐derived growth factor and tumor necrosis factor signaling, and cell adhesion molecules, all of which were previously found to be positively associated with disease. Using a reverse engineering approach, the same group reconstructed the regulatory network based on the co‐expression data in gingival tissues and subsequently interrogated the transcription factor or master regulator‐target gene interactions to identify periodontitis‐specific pathways.^10^ Of all the differentially regulated pathways, those related to the immune response and immune system development were the most frequently enriched. For example, B‐cell development, leukocyte extravasation signaling, phosphoinositide 3‐kinase signaling in B lymphocytes, B‐cell receptor signaling, and granulocyte adhesion and diapedesis were all among the top 10 most commonly enriched master regulator‐associated pathways. Importantly, some of the major differentially regulated pathways reported in those 2 studies^10^, ^14^ have also been identified in other independent studies^30^. For example, pathways involved in cytokine and chemokine activities, B‐cell receptor signaling, and defense and immunity proteins in both innate and adaptive immune responses, were reported to be among those most upregulated in periodontitis gingival tissues when assessed using RNA sequencing.^30^

However, novel pathways, or genes that have not been previously associated with periodontitis, emerged in those “hypothesis‐generating” transcriptome studies. For example, molecules participating in epithelial function and barriers were downregulated in gingival tissues affected by periodontitis. Demmer et al^14^ found that the desmocollin 1 gene, which encodes a calcium‐dependent glycoprotein primarily expressed in epithelial cells and is required for cell adhesion and desmosome formation, was the gene most significantly downregulated in periodontitis, with a transcription level of about one‐quarter of that of disease‐free controls. The expression of most keratin family genes, such as keratin 1, keratin 2, and keratin 3, was also significantly suppressed in diseased gingival tissue at the genomic scale. The cytokeratin family proteins that are expressed most abundantly in the upper spinous layer of the epithelium play a critical role in the establishment of the epithelial barrier.^31^ Desmocollin 1, keratin 1, and keratin 27, together with late cornified envelope protein 6A, another epithelial structural protein‐coding gene, were all independently reported in another study to be among the most downregulated transcribed genes in diseased gingival tissues.^30^ The downregulation of those epithelial homeostasis‐associated genes in periodontitis may reflect a compromised gingival or mucosal epithelial barrier, which enables easier penetration of plaque pathogens or their metabolites into the connective tissue compartment. Those infiltrated toxins or bacteria can directly induce inflammation in the subepithelial region and accelerate alveolar bone loss. Therefore, a decrease in epithelial barrier defense, mediated through an inhibition of those epithelium specfic gene expression constitutes an important, but not well defined mechanism for periodontal disease pathogenicity. Another common finding from transcriptome studies is upregulation of the interleukin‐17 pathway in periodontitis gingival tissues.^14^, ^30^ Interleukin‐17 is the hallmark cytokine primarily secreted by T helper 17 lymphocytes and other immune cell populations. The homeostatic activity of interleukin‐17 signaling in epithelial cells plays a protective role against pathogen invasion by recruiting adequate neutrophils to the battleground at the mucosal surface.^32^, ^33^ However, excessive secretion of interleukin‐17 has been pathologically linked to a range of inflammatory mucocutaneous diseases, such as psoriasis, lupus erythematosus, and ulcerative colitis,^34^, ^35^, ^36^ and increased numbers of interleukin‐17‐expressing cells are frequently encountered in those diseases. The role of interleukin‐17 in oral diseases is complex. The absence of interleukin‐17 activity severely compromises the antimicrobial defense mechanism. For example, patients with mutations that disrupt interleukin‐17 signaling are prone to fungal infection.^37^ Interleukin‐17 receptor A null mice are more susceptible to oropharyngeal candidiasis caused by the commensal fungus *Candida albicans* than T helper 1‐deficient mice.^38^, ^39^ However, exaggerated production of interleukin‐17 was also found in gingival tissues affected by periodontitis.^29^ Therefore, the homeostasis of this key cytokine, which balances interleukin‐17‐mediated mucosal defense and inflammatory response, is indispensable for maintaining periodontal health.

Transcriptome analysis at the genomic scale was also performed in gingivitis, a reversible inflammatory state that can be induced experimentally and safely in human participants. Employing a well‐established stent‐induced biofilm overgrowth model in human subjects,^40^ 2 research groups have characterized the stage‐specific gene expression profile paralleling the clinical induction and resolution phases of gingivitis.^12^, ^13^ By performing a microarray analysis on RNA samples isolated from gingival biopsy tissues at the baseline (day 0), induction (day 28), and resolution (day 35) phases, Offenbacher et al^13^ confirmed that the immune response was the most significantly activated biologic thematic pathway in experimental gingivitis. Transcription of genes associated with chemotaxis and transendothelial migration of leukocytes (interleukin‐8, C‐C motif chemokine ligand‐4, C‐C motif chemokine ligand‐5, C‐X‐C motif chemokine ligand‐3, C‐X‐C motif chemokine ligand‐2, C‐X‐C motif chemokine ligand‐12, and integrin subunit beta‐2), innate immunity activation (toll‐like receptor‐7, toll‐like receptor‐10, interleukin‐1A, and interleukin‐1B), and T‐cell activation (TCR gamma alternate reading frame protein, protein tyrosine phosphatase nonreceptor type 22, and cell receptor alpha locus), were all significantly increased during induction of gingival inflammation and decreased below the baseline in resolution. Transcription of genes associated with host‐microbe interactions and B‐cell activation exhibited a similar expression pattern. In a similar gene ontology analysis of a separate study, Jönsson et al^12^ also found that inflammation‐associated pathways, including leukocyte transendothelial migration, cell adhesion, antigen processing, and presentation, were among the most significantly activated biologic processes in both the induction and resolution phases, but in different directions. To a large extent, these transcriptomic findings at the molecular level echo the previously described histologic observations of the initial and early stages of gingival lesions.^41^, ^42^ However, unexpected novel findings emerged from those studies. For example, in the Offenbacher et al 13 study, neural processes were identified as the second most dominant cellular activation pathway. Expression of the neural chemokine genes, prokineticin‐2, and Kallmann syndrome‐1 were all upregulated during induction of inflammation. The neural thematic pathway has a wide range of communication with other thematic pathways, including immune response, epithelial tissue, vasculature, and wound healing. The neural involvement in gingival inflammation may reflect an unappreciated role of neural processes in modulating inflammation at different stages. In the Jönsson et al^12^ study, pathways related to barrier function, such as formation of adherent junctions and tight junctions, were also among the most commonly activated biologic processes. Therefore, it appears that epithelial barrier activity is critically involved in the pathogenicity of both gingivitis and periodontitis.

## 3 MICROBIAL DYSBIOSIS IN PERIODONTAL DISEASE

The current periodontal microbiology theory proposes that infectious oral diseases are the result of a “dysbiotic” biofilm rather than the direct effect of specific pathogenic bacteria in the host.^43^ This theory suggests that microbial synergy among biofilm colonizers shapes and stabilizes a disease‐provoking microbial profile that disrupts equilibrium with the host, leading to a diseased state. Several lines of experimental evidence, mostly centered on the classic periodontal pathogen, *Porphyromonas gingivalis*, support this notion. *Porpyromonas gingivalis* is not a potent stand‐alone inducer of inflammation, and often contradictory host responses to *P. gingivalis* are observed in vitro and in vivo. For example, *P. gingivalis* lipopolysaccharide can antagonize toll‐like receptor 4, unlike other highly pro‐inflammatory lipopolysaccharides from most gram‐negative bacteria.^44^ Similarly, in the absence of commensal bacteria, *P. gingivalis* fails to induce periodontitis when used as a mono‐infection in germ‐free mice.^44^ The dysbiosis theory then hypothesizes that *P. gingivalis* acts like a “keystone” pathogen, which even in the presence of a small fraction of the microbial community can elevate the pathogenicity of biofilm bacteria by disrupting host‐bacteria homeostasis.^43^

In fact, results from major human microbiome studies align well with the microbial dysbiosis theory in periodontitis pathogenesis. The advent of 16S ribosomal RNA gene‐sequencing technologies has allowed the evaluation of phylogenetic relatedness among bacterial species. Diaz et al^15^ summarized that: (a) health‐ and periodontitis‐associated microbial communities differ; (b) there is more bacterial diversity in subjects with periodontitis (eg, more species phylotypes are enriched in periodontitis) than in subjects with periodontal health; (c) health‐associated species are not lost or replaced but rather are suppressed; and (d) periodontitis is associated with shifts in the species that numerically dominate subgingival communities rather than with de novo colonization by new species.

### 3.1 Dysbiosis in gingivitis

The oral microbial transitions from periodontal health to disease involve microbial successions and adaptations to changing environments. Such transitions are also influenced by the host response in the gingival sulcus.^45^ Transition of the microbial ecology from periodontal health to gingivitis is probably best characterized by the reversible nature of experimental gingivitis in response to the lack of plaque control.^9^ Recent 16S ribosomal RNA high‐throughput sequencing analyses have confirmed early findings regarding the shift from gram‐positive cocci to gram‐negative morphotypes (rods, filaments, and spirochetes). Kistler et al^46^ evaluated the changes of microbial community diversity and shifts in periodontally healthy subjects, 1 and 2 weeks after oral hygiene abstention. Significant shifts in the microbiome were recorded during this transition, with particular attention focused on the correlation between the composition of the microbiome and bleeding on probing scores. They found that the bacteria which were negatively correlated with bleeding on probing were predominantly aerobic and facultatively anaerobic gram‐positive cocci and rods, including members of the genera *Actinomyces*,* Rothia*, and *Streptococcus* (classic early colonizers of the tooth surface).^47^ The species that increased in relative abundance as gingivitis developed and showed a positive correlation with bleeding on probing were mostly gram‐negative taxa of the genera *Campylobacter*,* Fusobacterium*,* Lautropia*,* Leptotrichia*,* Porphyromonas*,* Selenomonas*, and *Tannerella* (mostly obligate anaerobes).

### 3.2 Dysbiosis in periodontitis

Most studies analyzing subgingival microbial profiles agree that patients with periodontitis harbor a different bacterial community than people with periodontal health, usually with a greater abundance and a more diverse range of taxa in disease.^48^, ^49^, ^50^, ^51^ Such a different microbial profile is usually a result of differences in the relative abundance of taxa shared by both periodontal disease and health; that is to say, it is not entirely caused by novel colonizers. Therefore, the microbial shift from periodontal health to the diseased state is more than likely a microbial succession process in which the proportion of existing species changes as new colonizers emerge, and in which the taxa associated with periodontal health are not replaced.^48^

While gram‐negative anaerobic species are significantly enriched with increased gingival inflammation, the species that are enriched in gingivitis are not exactly the same as those associated with periodontitis. For instance, Diaz et al^15^ reported that only about 20% of gingivitis‐associated taxa are simultaneously associated with periodontitis. Also, within periodontitis‐associated taxa, different “clusters” can be present, depending on the severity of the disease. For example, “Cluster A” (milder forms of periodontitis) is enriched with *Campylobacter*,* Corynebacterium*,* Fusobacterium*,* Leptotrichia*,* Prevotella*,* Tannerella*, and *Saccharibacteria* (formelry known as candidate division TM7) species, whereas “Cluster B” (more severe periodontitis) is characterized by an enrichment of “red complex” bacteria, *Filifactor alocis*,* Treponema* spp., and *Fretibacterium* spp., as well as other species strongly associated with periodontitis severity.^51^

The shift in composition of the microbial community from periodontal health to periodontitis supports the concept that the host inflammatory or immune defense state has an impact on subgingival colonization. Griffen et al^49^ reported that, compared with healthy sites of periodontitis‐free subjects, the subgingival bacterial species from deep pockets of patients with periodontitis were enriched with 123 species that were significantly more abundant in disease, while in healthy sites only 53 species were significantly more abundant. This may reflect that the subversion of host defenses from health to disease permits colonization with larger numbers of bacterial species, especially those nonpathogenic commensals that favor pathogen growth. They also observed that shallow pockets from patients with periodontitis share a preponderance of disease‐associated species, as identified in deep probing pockets. This also strengthens the argument that host‐related factors, such as genetics, play an important role in influencing microbial community profiles.

The genetic background of patients plays a role in determining subgingival bacterial colonization. For example, patients with leukocyte adhesion deficiency‐I with a genetic defect of cluster of differentiation 18 (CD18) or integrin beta chain‐2 usually develop generalized periodontitis, clinically mimicking aggressive periodontitis at a young age.^52^, ^53^ However, the microbial composition of subgingival plaque in patients with leukocyte adhesion deficiency‐I is different from that in patients with either chronic or aggressive periodontitis. Aggressive periodontitis. In patients with leukocyte adhesion deficiency‐I periodontitis, the complexity of plaque microbial communities was actually reduced, while the diversity of such communities in patients with periodontitis, but with no known genetic defects, was usually increased compared with patients with periodontal health.^48^, ^54^, ^55^ In addition, analysis of plaque samples from patients with leukocyte adhesion deficiency‐I periodontitis demonstrated that *Aggregatibacter actinomycetemcomitans*, a species that is usually present in patients with aggressive periodontitis who do not have a known genetic or immune defect, was usually undetectable or only detected at a very low level. Interestingly, several species that are not usually associated with either chronic or aggressive periodontitis were uniquely present in patients with leukocyte adhesion deficiency‐I periodontitis. For example, *Pseudomonas aeruginosa*, which is not associated with periodontal disease but is an infectious agent in immunocompromised patients, was readily detected in patients with leukocyte adhesion deficiency‐I.^55^, ^56^ This antibiotic‐resistant bacterium, which can survive in hostile environments, may influence the formation of a microbial community that is unique to patients with leukocyte adhesion deficiency‐I periodontitis. Other colonizers specific to leukocyte adhesion deficiency‐I include *Leptotrichia* spp. that are not usually associated with common periodontitis. The genetics‐determined inflammatory state of patients with leukocyte adhesion deficiency‐I may have an impact on the leukocyte adhesion deficiency‐I‐specific microbial colonization. One unique inflammation signature of leukocyte adhesion deficiency‐I periodontitis is the uninhibited production of interleukin‐17 in local gingiva by increased numbers of T helper 17 cells.^29^ It is likely that the excessive secretion of interleukin‐17 in patients with leukocyte adhesion deficiency‐I periodontitis, who also harbor a genetic mutation in integrin beta chain‐2, promotes a dysbiosis that is distinct from the microbial communities associated with common periodontitis.

### 3.3 Dysbiosis and metabolic signatures associated with periodontitis

A DNA sequencing‐based profiling approach that explores the microbial community cannot readily provide information about whether the identified bacteria are metabolically active or even alive or dead, as the sequencing data do not measure the biologic activities of the bacteria. To address this caveat, Marchesan et al^19^ performed an analysis of microbiome microarray data and metabolite data from saliva by liquid chromatography‐mass spectrometry and gas chromatography‐mass spectrometry, and assessed the relationships among plaque microbial composition, salivary bacterial metabolites, and periodontal disease phenotypes in a well‐established stent‐induced biofilm overgrowth clinical model. Several newly identified putative periodontal pathogenic species in *Synergistetes* and *Treponema* phyla were significantly associated with periodontitis parameters.^57^, ^58^, ^59^ *Synergistetes* spp. were also associated with 2 novel dipeptides: cyclo(‐Phe‐Pro) and cyclo(‐Leu‐Pro). In addition to quorum‐sensing molecules, these metabolites possess bacteriolytic activity and therefore inhibit the growth of certain bacteria in biofilm.^60^, ^61^, ^62^ It is hypothesized that high levels of those cyclodipeptides promote dysbiosis by thwarting commensal bacteria and favoring the overgrowth of *Synergistetes* bacteria, which are clinically associated with severe periodontal disease. Another study reported using ribosome RNA‐sequencing to access aggressive periodontitis‐associated subgingival microbial composition and metatranscriptomic RNA‐sequence data to analyze microbial gene‐expression profiles from metabolically active microorganisms in the same plaque samples. The results showed that the disease‐associated microbial communities had overall significantly fewer species (alpha‐diversity) and less dispersed distribution (beta‐diversity) in diseased pockets than in periodontal health‐associated sites.^63^ Genes involved in conserved metabolic pathways, such as lysine fermentation to butyrate, histidine catabolism, nucleotide biosynthesis, and pyruvate formation, were consistently upregulated in diseased sites, and probably contribute to the disease process. The sequencing data also suggest that although subgingival plaque‐colonizing bacteria usually present high interindividual variability, the metabolic activities by colonizers are conserved because of the convergence of metabolic processes from diverse species of bacteria in plaque. This study also highlights the keystone role of *Fusobacterium nucleatum* in subgingival plaque. Although the plaque bacteria colonizing pockets in periodontitis and sites with periodontal health comprised similar percentages of *F. nucleatum*, the metabolic shift toward an enriched lysine‐derived butyrate from periodontal health to periodontitis, may establish a favorable environment for dysbiosis and promote disease activity.^64^, ^65^

Most microbiome studies use cross‐sectional data to compare the microbial profile of patients with periodontitis or diseased pockets with that healthy controls. However, such association data neither reveal the dynamic changes of microbial communities as the disease progresses nor do they provide strong evidence to suggest a causal relationship between certain pathogens and periodontal disease. To address this issue, Yost et al^66^ performed metagenomic and metatranscriptome analyses to characterize the bacterial profile and molecular activity signature associated with active periodontitis progression in a patient cohort. For these analyses they collected plaque samples at baseline and at follow‐up visits, and compared the microbial compositional and transcriptional changes between stable sites that did not have an increase of >1 mm in clinical attachment loss and sites that experienced disease progression, which was indicated by attachment loss of ≥2 mm between baseline and any of the follow‐up visits. Applying metagenomic shotgun‐sequencing, they reported that in the stable sites without disease progression very few changes were observed in the microbial community of the follow‐up samples in comparison with that at baseline. However, in progressing sites, more *Streptococcus* spp. significantly dominated the microbial profile at baseline, while more bacteria of the *Prevotella* genus and *Synergistetes* were present in sites showing disease progression at follow‐up visits. The high abundance of *Synergistetes* in periodontitis sites echoes findings from previous reports (50, 83). In their metatranscriptome analysis, transcripts related to oxidative stress response and pathogenesis‐associated gene ontology terms were enriched at the endpoint of disease progression. More specifically, transcriptional profiles from the red complex putative pathogens (*P. gingivalis*,* Treponema denticola*, and *Tannerella forsythia*) in sites showing disease progression demonstrated upregulation of genes involved in: proteolysis; transport of iron, lactate, sodium, and phosphate; the protein kinase C‐activating G‐protein coupled receptor signaling pathway; response to antibiotics; flagella biosynthesis; capsular polysaccharide biosynthesis; and conjugation. Synthesis of flagellae and capsule formation are clearly associated with invasion of tissue by bacteria and subversion of the host's defense, while the conjugation process facilitates communication among bacteria in biofilm through the horizontal transfer of genetic materials. By contrast, in stable sites without disease progression, no differential expression of genes was identified between samples collected at baseline and those collected at follow‐up visits. The disease‐progressing sites showed an over‐representation of genes related to cell motility, lipid A, and peptidoglycan synthesis, and the transport of amino acids, iron, and potassium, while nonprogressing stable sites did not. Unexpectedly, they found that bacteria not conventionally associated with periodontitis, such as *Streptococcus* genus, *Vellonella parvula*, and *Pseudomonas fluorenscens*, are active producers of large amounts of putative virulence factors. This finding may indicate the importance of the activities of commensal bacteria for supporting a dysbiotic microbial environment, or it may reflect the undefined pathogenic role of the early colonizers that had previously been associated with periodontal health.

## 4 GENETIC DETERMINANTS OF SUBGINGIVAL BACTERIAL COLONIZATION

The homeostasis at the mucosal surface is co‐determined by the colonizing bacteria and host immune response, underpinned by the host's genetics. A large amount of evidence has shown that resident microbes foster normal immune‐system development and mediate both innate and adaptive immune responses to achieve a symbiosis at different mucocutaneous niches of the host, such as skin, gut, and oral cavity. Convincing experimental data from germ‐free mice have provided clues as to how commensal bacteria “educate” immune systems by avoiding excessive immune reactions to achieve such a symbiosis. For example, polysaccharide A from *Bacteroides fragilis* in gut mucosa can exploit the host toll‐like receptor‐2 pathway to specifically suppress T helper 17 cell development. Such a *B. fragilis*‐associated toll‐like receptor‐2‐mediated inhibition of the T helper 17‐response promotes colonization of this commensal bacteria.^68^ By contrast, host genetic information shapes the composition of the microbiota residing in those niches by modifying immune responses. A good example that illustrates the host genetic influence on gut microbe colonization is from mice genetically deficient in the toll‐like receptor‐5 gene,^69^ which exhibit hallmark features of metabolic syndrome. Recipient wild‐type, germ‐free mice that were transferred with gut microbiota collected from those toll‐like receptor‐5‐deficient mice also developed many features of metabolic syndrome. Therefore, the colonization of gut biofilm by pathogenic bacteria is selective and takes place under the pressure of genetics‐mediated host immune responses.

It has long been hypothesized that the host genetic variants affect the colonization of bacteria in the subgingival ecological niche. In addition to a hyper‐ or hypo‐inflammatory response, the variant‐mediated host response may also determine the composition and microbial load of subgingival bacteria aggregating in plaque biofilm. This genetic effect on subgingival bacterial colonization is well illustrated in mice with a specific ablation of genes mimicking the extreme loss‐of‐function genetic variants or humans with genetic defects. For example, mice deficient in the developmental endothelial locus 1 gene deficiency harbored a significantly higher oral bacterial count than age‐matched control mice, which had a qualitatively different oral microbial community.^70^ Leukocyte adhesion deficiency‐I patients with molecular defects induced by mutations in the integrin beta chain‐2 (integrin subunit beta‐2) gene exhibited a significantly greater bacterial load in subgingival plaque and presented a unique disease‐associating composition of microbes, such as enrichment of *Treponema* spp., *Saccharibacteria* phylum, *Porphyromonas endodontalis*,* P. aeruginosa*,* Leptotrichia* spp., and *Scardovia wiggsiae*.^55^ These results indicate that microbial colonization and pathogenicity are profoundly affected by genetic determinants.

### 4.1 Genetic determinants of bacterial colonization by a candidate‐gene association approach

The “inside‐out” relationship, in which the host's intrinsic genetic factors affect microbial colonization at the gingival surface, has been explored using a candidate gene approach that links several preselected risk genetic variant markers to specific periodontal pathogens in subgingival microflora. Inconsistent results arose from those studies. Since Kornman^71^ reported that a positive composite haplotype in the interleukin‐1 gene cluster (rs1800587 within the interleukin‐1A locus and rs1143634 within the interleukin‐1B locus) was significantly associated with a more severe clinical disease phenotype in nonsmokers, different groups have explored the relationship between this composite haplotype and subgingival pathogenic bacterial species.^72^, ^73^, ^74^, ^75^, ^76^ Using a DNA‐DNA checkerboard hybridization approach, Socransky et al^72^ found that significantly more people with a positive composite haplotype in the interleukin‐1 gene cluster harbored bacteria from the red complex (including *T. denticola* and *T. forsythia*) and the orange complex (including *F. nucleatum* subspecies, *Campylobacter* spp., and *Streptococcus constellatus*) with mean bacterial counts above the threshold level, unlike those without this haplotype. They further concluded that the microbial burdens of those bacteria from deep periodontal pockets were significantly higher in carriers of the composite haplotype than in noncarriers.^72^ They proposed that the composite interleukin‐1 gene cluster induced excessive inflammation through upregulation of cytokines such as interleukin‐1beta, creating a favorable niche for overgrowth of those red and orange complex bacteria. Part of this inflammation‐induced pathogen‐supporting environment is mediated by an increased volume of gingival crevicular fluid and a breakdown of adjacent epithelial tissues, which provide nutrients to “feed” those pathogenic bacteria.^72^, ^77^ Schulz et al^73^ found that the risk for positive detection of subgingival *A. actinomycetemcomitans* was increased twofold in carriers of the interleukin‐1 gene composite haplotype, although they found no association between this haplotype and severe periodontitis. However, other research teams could not detect a relationship between the positive interleukin‐1 gene composite haplotype and periodontal pathogen colonization. Krátká et al^74^ reported no significant association between this haplotype and *A. actinomycetemcomitants* in the subgingival plaque from patients with aggressive periodontitis. Paradoxically, by quantifying subgingival microbial pathogens with the same DNA‐DNA checkerboard in a population of 151 subjects undergoing regular periodontal supportive therapy, Agerbaek et al^75^ reported that patients negative for the interleukin‐1 composite haplotype harbored almost twice as much of the red complex pathogen *P.* *gingivalis* and the orange complex pathogens *Eubacterium nodatum*,* Streptococcus anginosus*, and *A. actinomycetemcomitans*, than carriers of this haplotype. In an ethnically mixed population of 292 subjects, a positive *interleukin‐1* composite genotype was not associated with a higher detection rate of red complex bacteria or *A*. *actinomycetemcomitans* in the pocket biofilm of patients with or without periodontitis.^76^ A recent meta‐analysis failed to detect a significant association between composite interleukin‐1 gene variants and those potential pathogenic bacteria, including *P. gingivalis*,* T. forsythia*, and *A. actinomycetemcomitans*.^78^

Several other alleles suggestive of periodontitis risk, within genes encoding proteins involved in the inflammation process, have also been explored in association with certain subgingival bacteria. For example, by genotyping the interleukin‐6 single nucleotide polymorphism rs1800795 in pool of patients with untreated generalized aggressive periodontitis, Nibali et al^79^ reported that significantly more patients homozygous for the guanine allele (GG) than patients with heterozygosity (GC) or homozygosity for the cytosine (CC) allele genotype harbored *A*. *actinomycetemcomitans* with or without the simultaneous presence of *P. gingivalis* in their deepest periodontal pockets. Individuals from a homogenous Indian population with the GG allele genotype of interleukin‐6 exhibited a significantly higher pathogen load in their subgingival plaque than those with other genotypes.^80^ Homozygosity for the C allele of another variant site at rs2069825 within the interleukin‐6 gene promoter region has been demonstrated as significantly associated with the presence of *A*. *actinomycetemcomitans* (odds ratio = 2.71, 95% confidence interval: 1.51‐4.83) and *P. gingivalis* (odds ratio = 1.98, 95% confidence interval: 1.09‐3.60) in the subgingival biofilm.^81^ By applying binary logistic regression analysis, Schulz et al^82^ identified that a variant A allele of adeine‐guanine (AG) or homozygosity for the A allele (AA) genotype in the rs1800629 locus within the tumor necrosis factor‐alpha locus was significantly associated with *Prevotella intermedia* subgingival colonization after adjusting for confounding variables. However, in a separate report, Trombone et al found no associations among the same tumor necrosis factor‐alpha‐variant genotypes and the presence of *A*. *actinomycetemcomitans* or individual or combined red complex bacteria.^83^ Meta‐analyses linking those non‐interleukin‐1 genetic variants with subgingival pathogens are not available because of data heterogeneity in ethnicities or medical history in those studies.^78^

### 4.2 Genetic determinants of bacterial colonization using a genome‐wide association approach

The limited success of the candidate gene approach is usually confined by the lack of power inherent to the small sample size frequently seen in those studies, increased risk of bias because of the case‐control design, which is based on care‐seeking rather than a population‐based method, and high population heterogeneity with an absence of stratification.^78^, ^84^ Recently, several genome‐wide association studies carried out in well‐defined populations have shed light on the genomic underpinning of clinical disease traits or phenotypes. This unbiased analytical approach has identified several suggestive microbial pathogen associated genetic loci.^23^, ^25^, ^67^ To date, most of the genome‐wide association studies on subgingival microbial colonizers have been based on data obtained from a monitored community‐dwelling Caucasian population participating in the Atherosclerosis Risk in Communities study.^85^ Although no single nucleotide polymorphism variant passed the conventional genome‐wide association study threshold (*P* = 5 × 10^−8^), Divaris et al^23^ identified several suggestive loci (*P* < 5 × 10^−6^) in 1020 participants that were associated with high red complex, orange complex, and *A. actinomycetemcomitans* colonization profiles generated by DNA‐DNA checkerboard hybridization. Those loci are either located within structural or promoter regions of genes, or are dispersed in the intergenic regions. For example, the G allele at the rs11800854 locus was associated with a high microbial burden of both red complex (*P. gingivalis*,* T. forsythia*, and *T. denticola*) and *A. actinomycetemcomitans* with an odds ratio of 12.3 (*P* = 2.8 × 10^−7^). This variant site is in the promoter region of potassium channel subfamily K member 1, a potassium channel gene whose overexpression is linked to heart failure.^86^ The same single nucleotide polymorphism variant is also close to mitogen‐activated protein kinase kinase kinase 21, a gene‐encoding mixed lineage kinase 4 that negatively regulates lipopolysaccharide‐mediated toll‐like receptor‐4 signaling. 87 Another variant at rs1932040 (A/G) that is associated with a high level of colonization with bacteria of the orange complex is located in an intergenic area encompassed by the chloride intracellular channel protein 5 gene and the runt‐related transcription factor 2 gene, which encodes an osteogenic marker that is involved in periodontitis.^88^ Other suggestive lead single nucleotide polymorphism variants are located in proximity to genes encoding proteins involved in inflammation and the immune response (eg, interleukin‐33, vesicle‐associated membrane protein‐3), transcriptional factors involved in apoptosis and differentiation (eg, Fos proto‐oncogen [AP1 transcription factor subunit]), and cell stress response (eg, jun dimerization protein 2).^89^, ^90^, ^91^ The effect of most of the suggestive loci on those pathogen communities had at least the same direction in an African‐American population with a smaller sample size (*n* = 123), as reported in a separate analysis.^23^ Subsequently, applying a gene‐centric and gene set‐enrichment association analysis to the same data set for the 1020 Caucasian atherosclerosis risk in communities population, Rhodin et al^67^ demonstrated that both potassium two pore domain channel subfamily K member‐1 and DAB2 interacting protein genes had a statistically significant association with *P. gingivalis* colonization. Notably, the DAB2 interacting protein is a signaling adaptor molecule implicated in the innate immune response. Ablation of the gene encoding DAB2 interacting protein led to increases in plasma inflammatory cytokine levels and exacerbated atherosclerotic lesions in DAB2 interacting protein gene knockout mice.^92^

**Figure 1:**
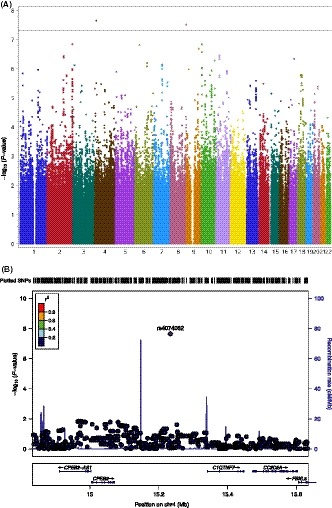
Both of the single nucleotide polymorphisms rs4074082 and rs9772881 are significantly associated with periodontal complex trait 3 (PCT3 or *Aggregatibacter* *actinomycetemcomitans* trait) (*P* < 5 × 10^−8^). Manhattan plot for PCT3 (A) and locuzoom view of rs4074082 (B) are presented

Under the hypothesis that periodontitis is comprised of a mixture of distinct biologic conditions with similar clinical phenotypes or disease presentation, Offenbacher et al^25^ defined several periodontal complex traits using principal component analysis combining clinical disease measurements, gingival crevicular fluid interleukin‐1beta level, and the microbial burden of eight putative periodontal pathogens, and identified a number of genetic determinants associated with those traits in the Caucasian atherosclerosis risk in communities population. Periodontal complex trait 1, which explains the greatest variance in the principal component analysis model, is highlighted with an even loading of almost all 8 pathogens, referred to as a Socransky trait. This trait may possess the microbial community structure associated with the most common form of periodontal disease. Periodontal complex trait 3, which is characterized by a high loading of interleukin‐1beta and *A. actinomycetemcomitans*, is referred to as the *A. actinomycetemcomitans* trait, while periodontal complex trait 5 is denoted as the *P. gingivalis* trait because of the high loading of *P. gingivalis* in the principal component analysis model. Several genome‐wide statistically significant genetic markers have been determined for each periodontal complex trait. For example, candidate single nucleotide polymorphism rs1633266 within interferon gamma inducible protein‐16 and absent in melanoma‐2 genetic loci is significantly associated with the Socransky trait (periodontal complex trait 1). Both genes are involved in the intracellular processing of foreign DNA and the immune response. Lead candidate single nucleotide polymorphism rs4074082 (*P* = 2.2 × 10^−8^) and rs9772881 (*P* = 3.1 × 10^−8^) are both significantly associated with the *A. actinomycetemcomitans* trait (periodontal complex trait 3) trait (Figure 1A). Single nucleotide polymorphism rs4074082 is close to the complement C1q tumor necrosis factor‐related protein‐7 gene, which plays a role in neural inflammation (Figure 1B). The expression of complement C1q tumor necrosis factor‐related protein‐7 was found to be downregulated in amyotrophic lateral sclerosis, a neurodegenerative disorder in which abnormal inflammatory response is involved as part of the etiology.^93^ rs9772881 is approximate to the TSNARE1 gene locus. Single nucleotide polymorphism variant rs6488099, which is significantly associated with the *P. gingivalis* trait (periodontal complex trait 5), is located in the plakophilin‐2 locus, which encodes a structural protein component of desmosomes that regulate oral epithelial barrier activities. However, whether this particular single nucleotide polymorphism variant leads to plakophilin‐2 gene gain‐of‐function or loss‐of‐function is unknown. Prior to genome‐wide association study findings, the plakophilin‐2 molecule had not been elucidated in periodontal disease. Therefore, our group started to assess the novel role of the plakophilin‐2 gene in periodontal disease by focusing on clinical gingival biopsy samples from human periodontitis subjects and in vitro analysis of epithelial plakophilin‐2 gene function in response to *P. gingivalis* challenge. Unpublished data showed that downregulation of the plakophilin‐2 gene the transcriptional level was associated with chronic periodontitis, and plakophilin‐2 molecules were degraded by cysteine proteases secreted by *P. gingivalis*. The loss‐of‐function, therefore, was favored in the pathogenic role of plakophilin‐2 gene variants in periodontal disease. Additionally, a short hairpin RNA interference experiment delineated the impact of plakophilin‐2 gene loss‐of‐function in epithelial cells. In knocking down the plakophilin‐2 gene leads to inhibition of cell proliferation and spreading, which suggests a weak gingival epithelial barrier that may allow easier invasion by *P. gingivalis*. Therefore, we speculate that patients carrying different plakophilin‐2 gene single nucleotide polymorphism variants might respond differently to microbial challenges, especially with *P. gingivalis*. Further studies will focus on the molecular mechanisms of how the identified variants affect gene activities and modify host susceptibility to periodontal disease.

**Figure 2:**
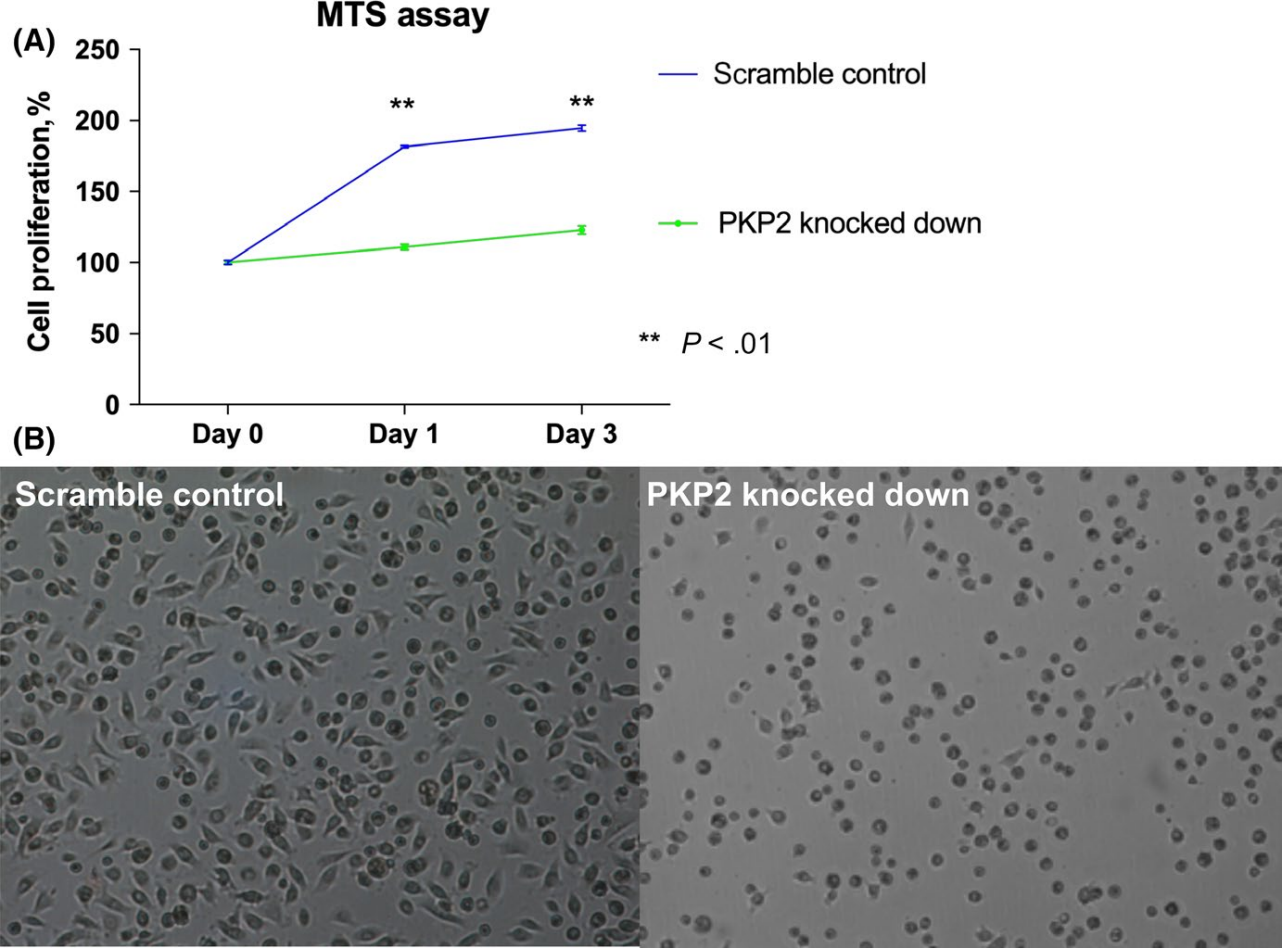
Plakophilin 2 (*PKP2*) loss‐of‐function inhibits cell proliferation and cell‐to‐cell contact. *PKP2* knockdown in primary gingival epithelial cells significantly hindered cell proliferation, as assayed by the MTS assay (A) and impaired cell spreading with increased gaps among cells 1‐h post‐seeding (B)

### 4.3 Validation of candidate genetic markers

The identified variant loci or candidate gene targets that are associated with either specific bacterial pathogens or the subgingival plaque microbial community from the aforementioned coronary cohort by genome‐wide association study need to be validated in other populations. Independent genome‐wide association investigations have also identified periodontitis‐associated loci close to genes that were reported in the genome‐wide association study in the atherosclerosis risk in communities population. For example, the variants encompassing the neuropeptide Y gene, one of the four target genes which met the gene‐centric statistical significance threshold in participants of the atherosclerosis risk in communities study, have also been marked by the genome‐wide genotyping data from a separate community‐dwelling adult population in a US and a German population.^24^, ^94^ However, genome‐wide analyses on either the presence or the counts of certain subgingival plaque pathogens other than in the atherosclerosis risk in the communities population are not available.

**Figure 3:**
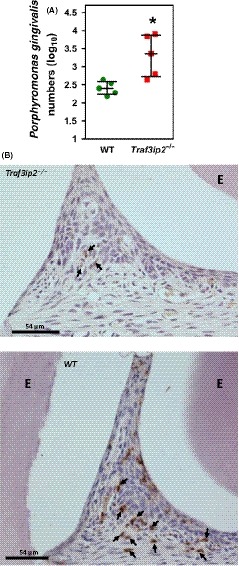
TRAF3 interacting protein‐2 (*Traf3ip2*)^*−/−*^ mice harbored more *Porphyromonas gingivalis A7436* and had less neutrophil infiltration than age‐matched wild‐type (WT) control animals. Mice were orally inoculated with *P. gingivalis A7436* for 14 d. Plaque samples were collected 2 d after the last inoculation and *P. gingivalis* was quantified by real‐time quantitative PCR against a standard. (A) Significantly more *P. gingivalis* was present in oral plaque from *Traf3ip2* ^*−/−*^ mice than in oral plaque from wild‐type controls (*P = .008 Mann‐Whitney test). (B) Fewer neutrophils (leukocyte antigen‐6‐gene‐positive cells stained by immunohistochemistry, as indicated by arrows) were present in the gingival tissue of *Traf3ip2* ^*−/−*^ mice (upper panel) than in the WT controls (lower panel); E, enamel

Conventional or gene‐centric enrichment genome‐wide association study analyses pinpointed genetic markers or candidate gene loci that have not been previously associated with periodontal disease. Before fine‐tuning the variant effects of those periodontal phenotype‐associating genetic markers in cells or in vivo using animal models, it is critical to understand the role of single nucleotide polymorphism‐affected novel genes in periodontal disease. Bioinformatics predicts that certain single nucleotide polymorphism variants, especially coding nonsynonymous ones in exons, will have a deleterious effect on the affected gene. Although deleting the genes of interest in mice can only simulate the rare extreme variant detrimental effect, which totally obliterates the gene activity, this knockout approach provides the initial insight into how those novel candidate genes mechanistically modulate the host inflammatory response in periodontal disease. According to the gene‐centric analysis mentioned above, the combined genetic variants within TRAF3 interacting protein‐2 (also called *CISK* or *ACT1*) gene locus are significantly associated with the *A. actinomycetemcomitants* trait (periodontal complex trait 3) at the gene level (*P* = 1.36 × 10^−6^).^25^ TRAF3 interacting protein‐2 is a critical adaptor protein for almost all of the interleukin‐17 receptor family members. Several independent genome‐wide association studies have repeatedly reported a coding nonsynonymous variant of rs33980500 within the first exon of the TRAF3 interacting protein‐2 gene as a risk allele for psoriasis or psoriatic arthritis. This variant induces an amino acid change from aspartic acid to asparagine and abolishes the downstream partner binding activity in the interleukin‐17 signaling pathway. However, the role of the TRAF3 interacting protein‐2 gene in periodontal disease has not yet been described. The “A” variant allele of rs33980500 exhibited a high linkage disequilibrium (*D*′ = 1, *R* ^2^ = 0.88) with the top coding nonsynonymous variant “T” variant allele of rs13190932 that was identified by our atherosclerosis risk in communities genome‐wide association study (*P* = 8.43 × 10^−7^) for periodontal disease. Using TRAF3 interacting protein‐2‐null mice that mimic the loss‐of‐function of the extreme damaging variant effect, we observed that TRAF3 interacting protein‐2^*−/−*^ mice delayed the clearance of the pathogen *P. gingivalis* after oral pathogen challenge (Figure 3A). Significantly more *P. gingivalis* was present in TRAF3 interacting protein‐2‐null mice than in control wild‐type mice, in the plaque samples collected after oral infection. Such a delay in clearance of *P. gingivalis* was associated with lower neutrophil infiltration of gingival tissues in knockout mice (Figure 3B). Therefore, a weakened epithelial defense because of inefficient neutrophil recruitment in those interleukin‐17 adaptor knockout mice permits colonization with a higher level of pathogens in the mouse oral cavity. This finding is supported by a genetic report demonstrating that patients carrying this TRAF3 interacting protein‐2 single nucleotide polymorphism variant of rs33980599 are susceptible to chronic mucocutaneous candidiasis in the tongue, oral mucosa, and skin caused by *C. albicans* infection.^37^ In addition, our unpublished data indicate that TRAF3 interacting protein‐2^*−/−*^ mice were more susceptible to *P. gingivalis*‐induced alveolar bone loss. These results provide evidence to support that the homeostatic TRAF3 interacting protein‐2‐mediated interleukin‐17 pathway strengthens the gingival mucosal defense or barrier activity by eliminating invading pathogens through engaging neutrophils. Several follow‐up in vitro and in vivo studies are currently ongoing to validate the biologic relevance of other variants or candidate genes identified from genome‐wide association studies.

It is critically important for the genetic variant markers identified through an essentially statistical approach to be verified biologically in vivo. The biologic validation of a genome‐wide association study candidate genetic locus is exemplified by a follow‐up investigation employing interleukin‐37 gene variant‐carrying mice in a ligature‐induced murine periodontitis model.^26^ The absence of a homolog of the human interleukin‐37 gene makes mice an appealing tool with which to assess the single nucleotide polymorphism variant effects identified in humans because one can directly observe the expression and function of variant‐carrying human interleukin‐37 knock‐in genes in disease models rather than mutating the counterpart nucleic acids in an innate murine homologue gene.^95^ A nonsynonymous single nucleotide polymorphism of rs3811046 within exon 2 of the interleukin‐37 gene was discovered to be significantly (*P* = 3.3 × 10^−22^) associated with a high interleukin‐1beta phenotype, which was defined as the top quartile of gingival crevicular fluid interleukin‐1beta level in the atherosclerosis risk in communities population. The integral interleukin‐37 cytokine possesses anti‐inflammatory activity.^96^, ^97^, ^98^ The minor G variant allele of rs3811046, with a frequency of 0.3 in Caucasians and polymorphism of rs3811047, which is in tight linkage disequilibrium with rs381146 (*D*′ = 1.0, *r* ^2^ = 0.98), is predicted to compromise interleukin‐37 protein by altering the key amino acids, V31G and A42T, respectively. Levels of interleukin‐1beta and interleukin‐8 in gingival crevicular fluid are significantly elevated among subjects with homozygous variant alleles for both single nucleotide polymorphisms compared with nonvariant‐carrying individuals in a separate population. In addition to replication in human participants, interleukin‐37 transgenic mice carrying both rs3811046 and rs3811047 variants exhibited significantly more alveolar bone loss in a ligature‐induced murine periodontitis model than wild‐type interleukin‐37 transgenic mice. The bone marrow‐derived macrophages from variant‐carrying mice also secreted more interleukin‐6 and interleukin‐1beta cytokines upon challenge by lipopolysaccharide.^26^ Those biologic data proved that both variant loci within the interleukin‐37 gene promote a hyperinflammatory phenotype by upregulating local production of the cytokine, interleukin‐1beta, in gingival tissue that contributes to alveolar bone loss.

Preliminary results from other studies aiming to test the biologic relevance of several candidate genes suggested by atherosclerosis risk in communities‐based genome‐wide association study analyses have shown promising signs that those variant‐affected genes may impact the colonization of periodontal pathogens and induce dysbiosis.

## 5 FUTURE DIRECTIONS

**Figure 4:**
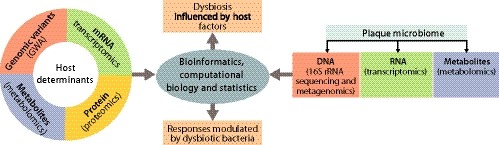
Global profiling or “‐omics” data sets obtained from the host and plaque biofilm are integrated through refined bioinformatics, computational biology, and statistics tools to evaluate the host's determinates on bacterial colonization in plaque biofilm and host responses to a dysbiotic microflora in the subgingival environment. GWA, genome‐wide association analysis; rRNA, ribosomal RNA

The sophisticated sequencing tools now available provide ample opportunities to predict host‐pathogen interactions at a global scale on the gingival surface. The metabolomic and proteomic work further adds functional dynamics to the interplay between the host and the subgingival microbiota. However, we are still at the infancy of comprehending those global changes of and interactions between the host response and plaque bacteria activities that determine an individual's susceptibility to periodontal disease and shape the disease's course. One of the challenges we are confronting is how to harmonize and integrate these “‐omics” profiling data to comprehensively understand periodontal disease processes. More refined data‐management tools, statistical and mathematical models, and bioinformatics are necessary for improved data integration. Such a holistic “multidimensional” approach allows investigators to assess the host's genetic impact on bacterial colonization in plaque biofilm and host responses to dysbiotic microflora in the subgingival niche (Figure 4). Future efforts are also required to replicate and biologically validate the candidate targets screened from “‐omic” data. For example, novel genomic markers suggested by the genome‐wide association studies from the atherosclerosis risk in communities population need to be replicated by other studies using microbial pathogens as a phenotypic component. The variant‐associated subgingival microbial community shifts and metabolic changes are largely unknown and need to be explored. The role of genes that have not been previously implicated in periodontal disease can be evaluated in vivo using transgenic mice in various periodontal bone loss models. In addition, candidate single nucleotide polymorphisms can be genomically engineered through the clustered regularly interspaced short palindromic repeats/CRISPR‐associated protein 9 (CRISPR/CAS9) system in cells and mice where the variant effects can be assessed.^99^

## 6 CONCLUSIONS

Global profiling data enable a comprehensive assessment of biofilm bacteria‐host interactions in periodontal disease. Indeed, the multidimensional information derived from these “‐omics” data sets confirmed certain findings from previous studies that have shaped our current understanding of disease processes. Nevertheless, novel single nucleotide polymorphisms, genes, pathways, metabolites, and bacterial species that have not been previously associated with periodontal disease are emerging. Those new candidate targets clearly require replication in other studies with a similar design but performed in different populations. More importantly, these novel disease‐associating markers need to be validated biologically in vitro and in vivo using animal models. In addition, more advanced bioinformatic tools are called for to harmonize these “big” data of different dimensions to advance precision medicine to measure an individual's risk for periodontitis and to provide the rationale for personalized periodontal therapies.
